# Effectively engaging African American and Latino communities on brain health and Alzheimer’s disease prevention

**DOI:** 10.1093/geront/gnaf232

**Published:** 2025-10-09

**Authors:** Daphne Delgado, Stephanie J Monroe

**Affiliations:** Health Equity, UsAgainstAlzheimer’s, Washington, District of Columbia, United States; The Wrenwood Group, Washington, District of Columbia, United States

**Keywords:** Health equity, Community health, Preventive health

## Abstract

Many have long believed that underrepresented communities are difficult to reach. UsAgainstAlzheimer’s (UsA2) understands communication methods and language used to reach White people may not be as effective when used to reach African Americans, Latinos, or other populations. It is therefore imperative that messages be personalized in such a way to be culturally relevant to and resonate with minoritized communities and that those messages be delivered by trusted members of the community, such as known health care providers, social workers, and community health workers. These trusted messengers often reside in and understand the communities they serve, which gives them a distinct advantage. Research has shown that nurses are some of the most trusted communicators of health information to their peers and the communities they serve. This article explores learnings from UsAgainstAlzheimer’s work, funded through the Centers for Disease Control and Prevention’s (CDC’s) National Healthy Brain Initiative cooperative agreement, including providing specialized training to nurses. It also addresses the impact of Alzheimer’s on African American and Latino communities and how to communicate action-oriented strategies to reduce the risk of developing Alzheimer’s in these communities.

## Introduction: Setting the context

Today, over 7 million Americans are living with Alzheimer’s disease and related dementias (ADRD), of which approximately two-thirds are women. Alzheimer’s is currently the seventh leading cause of death for all Americans. African American people are at least two times as likely to develop Alzheimer’s as White people, and Latino people are 1.5 times as likely to develop Alzheimer’s as White individuals (Alzheimer’s Association, 2025). For people living with ADRD, the inequities are profound and likely to continue to grow exponentially if left unaddressed.

In 2020, the total economic impact for ADRD for African American and Latino adults reached almost $94 billion. By 2060, the economic impact of ADRD for African American and Latino adults will increase almost 15-fold to $1.4 trillion (about $4,300 per person in the United States in 2060), assuming the incidence of ADRD and current trends in population growth for African Americans and Latinos remain the same (Mudrazija et al., 2025).

Many of those providing unpaid care to individuals living with ADRD are and will continue to be forced to reduce their participation in the workforce or, worse still, leave the workplace entirely, resulting in the loss of income for their families and taxable income for states and the federal government. Unless we can effectively prevent, reduce, or more effectively treat those diagnosed with ADRD, the United States will face significant and even catastrophic economic, family, societal, and governmental impacts (Gaskin et al., 2013; Mudrazija et al., 2025).

In 2023, an estimated 48.3 million people self-identified as Black, making up 14.4% of the population of the U.S. population (Pew Research Center, 2025). In the same year, there were 65.2 million Hispanics in the United States, making up 19.5% of the total U.S. population (Pew Research Center, 2024). By 2030, the African American and Latino communities aged 65 years and older will grow 114% and 224%, respectively, compared to a 65% growth rate for Whites (Aranda et al., 2019). This trend foreshadows substantial growth in the number of cases of Alzheimer’s, particularly in underrepresented communities, as the number one risk factor for Alzheimer’s is advanced age. Despite their increased risk, African Americans and Latinos are less likely to receive an accurate or timely diagnosis from a health care provider, more likely to be diagnosed in the later stages of the disease when medications are less effective, and less likely to be represented in either pharmacological or non-pharmacological research for cognitive decline and dementia (Tsoy et al., 2021).

Given the increased risk and shifting age demographics of African Americans and Latinos, it is critical to better engage and mobilize these highly impacted, often marginalized communities that have historically faced discrimination and racism in health care as well as other areas. Messages and materials must be written by and communicated through relevant channels and trusted providers to ensure that individuals access to and have a willingness to engage, believe, and yes, implement key risk modification strategies. While many in these populations rely on physicians to deliver and interpret medical results, nurses have been repeatedly recognized overall as the most trusted professionals (Brenan and Jones, 2024). Therefore, it is vitally important that health care professionals, and in particular nurses, be well-equipped to communicate scientifically validated messages designed to address brain health across the lifespan and empower those at the highest risk to understand cognitive impairment and ADRD as public health issues.

In 2020, the Centers for Disease Control and Prevention (CDC) awarded UsAgainstAlzheimer’s (UsA2) a 5-year cooperative agreement (DP20-2003: The National Healthy Brain Initiative) to more effectively reach African American and Latino communities with information on why it is crucial for them to take charge of their brain health by adopting scientifically validated strategies (Livingston et al., 2024) shown to be effective in reducing risk factors associated with dementia.

## A note about racial and ethnic terminology used in this article

This paper, wherever possible, keeps the racial and ethnic terms used in source documents. When not referring from specific source documents, the authors follow U.S. Office of Management and Budget (OMB) Statistical Policy Directive No. 15 (SPD 15) definitions (Office of Management and Budget, 2024). For simplicity, this paper uses “African American” for the “Black or African American” and “Latino” for the “Hispanic or Latino” OMB definitions.

## Identifying issues and appropriate strategies

To begin the planning process, UsAgainstAlzheimer’s first conducted a landscape assessment and a series of focus groups with African American and Latino people to better understand gaps in resources and current knowledge of brain health and cognitive impairment.

In coordination with Dr David Marquez of the University of Illinois Chicago, UsA2 conducted a comprehensive landscape assessment to identify gaps in messages and resources and explore effective messaging strategies focused on Latinos and African Americans. The landscape assessment searched for relevant literature through six databases (PubMed, Web of Science, Scopus, CINAHL, Embase, and Medline via Ovid) and included a review of online resources and platforms, such as the CDC. The assessment was done in spring 2021 and most of the literature found was published between 2009 and 2020.

The landscape assessment specifically focused on peer-reviewed literature assessing (1) healthy behaviors related to cognitive impairment and ADRD; (2) health education related to cognitive impairment and ADRD; and (3) the overlap between cognitive impairment and ADRD and COVID-19 (no peer-reviewed articles were found on this topic). The review analyzed messages focused on the following: (1) minority-identified and minority-serving health providers, with a focus on nurses; (2) African American consumers (aged 35–75 years); and (3) Latino consumers (aged 35–75 years). Nurses were prioritized because they consistently rank as the most trusted profession (Brenan and Jones, 2024) and the 35–75 years age range was used because it coincides with middle and late age, where people are at most-risk for AD. The analysis also reviewed popular literature and governmental sources focused on materials designed to recruit African Americans and Latinos into Alzheimer’s clinical trials. The landscape analysis review was broken down into eight sections:

1. Perceptions of health behaviors and brain health among people of color
2. Cultural tailoring of brain health information to African Americans
3. Cultural tailoring of brain health information to Latinos
4. How to disseminate brain health information/messages in general to people of color
5. How to disseminate brain health information/messages to African Americans
6. How to disseminate brain health information/messages to Latinos
7. Programs to improve brain health in people of color
8. Gaps and recommendations from peer-reviewed and popular media sources for tailoring and disseminating brain health information to African American and Latino people

Focus groups were then held to discuss the top five gaps identified in the landscape assessment. Four virtual focus groups, moderated by Alzheimer’s Los Angeles and observed by UsAgainstAlzheimer’s, were conducted in the first year of the cooperative agreement in summer 2021. Two focus groups focused on members from the priority populations (one with nine African American participants, four women, and five men, aged 42–68 years from six states; the other with eight Latino participants, six women and two men, aged 47–70 years from four states). Another two focus groups focused on African American and Latino nurses, with nurses who were members of either the National Association of Hispanic Nurses (NAHN) and/or the National Black Nurses Association (NBNA)—one group included nine Black Nurses, seven women and two men, from six states and the District of Columbia; and the other group included nine Latino nurses, seven women and two men, from six states. Several nurses had backgrounds in military health care, neurology, psychiatry, mental health, and/or geriatrics.

After completing the landscape assessment and focus groups, UsA2 narrowed the identified challenges and barriers identified in community engagement literature to the following three issues:

*Issue 1: Clarify the meaning of “brain health.”* The focus groups conducted with members from the two priority populations revealed that communities understand the term “brain health” differently. To some, it does not resonate at all because they have never heard the term used by medical personnel. To others, it describes intelligence or mental health and thus creates additional barriers, confusion, and lack of trust. The World Health Organization (WHO) defines brain health as “the state of brain functioning across cognitive, sensory, social-emotional, behavioural and motor domains, allowing a person to realize their full potential over the life course, irrespective of the presence or absence of disorders” (World Health Organization, 2022). As of 2020, CDC defined brain health as “an ability to perform all the mental processes of cognition, including the ability to learn and judge, use language, and remember” (Wang et al., 2020). It was therefore essential to agree on a central definition that UsA2 would use for this work.

Given the audiences UsA2 hoped to connect with, the following definition was adopted: “Brain health refers to the overall well-being and optimal functioning of the brain, allowing a person to realize their full potential over the life course.”

*Issue 2: The need to reach African American and Latino communities with accessible messages written at an appropriate health literacy level.* The next challenge identified focused on how to reach African American and Latino communities with messages about brain health and its vital importance to the entirety of a body’s functions, not just cognition or memory. UsA2 identified the following factors as essential to engaging African American and Latino populations, as validated by findings in the landscape assessment: (1) selecting terminology that was generally understandable and relatable; (2) using visual graphics, depictions, and acceptable comparators that would help communities relate more easily to shared information; (3) identifying what was needed to design messages that would have the most significant potential to reach, resonate with, and impact the two priority populations of focus; (4) ensuring that the messages are culturally sensitive and written in a style and at a health literacy level that is easily understood; and (5) including relevant information that is compelling, engaging, and likely to incite sustainable behavior change in the recipient.

*Issue 3: The need to identify and empower individuals in the community who can best deliver evidence-based messaging*. Through the research process (literature assessment, focus groups, etc.), it quickly became evident that nurses are among the most trusted of all health care workers in minoritized communities. They are on the frontlines—seeing all types of patients from all walks of life. They are well-positioned to manage care teams and link clinical care with public health and social services.

## Goal setting and progress

UsAgainstAlzheimer’s set out two main goals to address the issues and gaps previously identified:

*Goal 1: Reduce disparities among African Americans and Latinos by increasing the number of minority-serving health providers who are familiar with brain health, ADRD, and cognitive impairment and who can provide tailored communications about these health issues to African Americans and Latinos in their communities.* The major gaps previously identified that informed UsA2’s work on Goal 1 were: (1) a lack of culturally tailored and accessible provider messages about ADRD health disparities, brain health, and risk reduction strategies and (2) the need to better equip the provider workforce, and especially nurses, with information on how to discuss ADRD with their patients.

*Goal 2: Increase awareness among the priority populations through tailored dissemination of population-level communications and resources to community-based organizations, minority-serving media, and digital platforms.* The major gaps previously identified that informed UsA2’s work on Goal 2 were: (1) a lack of culturally tailored messages about how health behaviors affect ADRD; (2) a lack of culturally tailored and accessible community messages about ADRD health disparities, brain health, and risk reduction strategies; and (3) a lack of knowledge about how to deliver those messages to the prioritized communities in meaningful and effective ways.

UsA2 implemented several strategies and initiatives that address one or both goals:

### Brain Health Equity Nurse Fellowship

In partnership with NAHN and NBNA, UsA2 created the Brain Health Equity Nurse Fellowship (Fellowship) to address several key challenges:

- African American and Latino people have higher levels of mistrust of the health care system than White people (Armstrong et al., 2007; Bazargan et al., 2021).
- Although nurses have been the most trusted profession in the United States for over 23 years (Brenan and Jones, 2024), they receive little to no formal education in ADRD or brain health, whether during nursing school or in post-professional continuing education opportunities (Wiese & Williams, 2018).

To better equip nurses with the information and training they need, the Fellowship established key objectives for the program:

- *Improve Knowledge*: Provide fellows with tailored continuing education on Alzheimer’s and related dementias to address brain health knowledge gaps.
- *Cultivate Leadership*: Provide fellows with the leadership skills and content needed to promote tailored brain health education among their peers and in their community.
- *Facilitate Networking*: Connect fellows to a national network of nurses and experts working at the intersections of brain health, public health, and health equity to create opportunities for collaboration and knowledge sharing.

**Figure 1.**
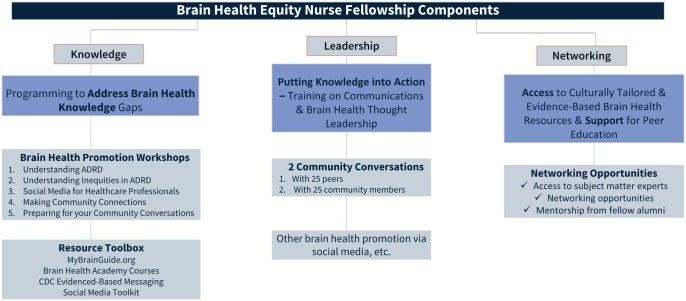
Brain Health Equity Nurse Fellowship components. ADRD = Alzheimer’s disease and related dementias.

The fellowship runs yearly cohorts, starting in 2022 (Cohort #1 was in 2022; #2 in 2023; #3 in 2024; #4 currently running in 2025). Before acceptance into the program, applicants must be members of NAHN and/or NBNA and go through a competitive application process. Applications are initially reviewed by NAHN and NBNA and final determinations are made by UsA2. All fellows are awarded a $2,000 stipend and over the course of the 9-month program are required to complete five virtual workshops, receive mentoring from an alumni mentor (component added during cohort #2), and lead at least one ADRD risk-reduction “Community Conversation” presentation with two audiences: (1) peers (broadly defined—nurses, social workers, community health works, physicians, etc.) and (2) lay community members (people living in the communities the fellows serve; component added during cohort #2). Fellows are expected to reach 25 people per audience. The mandatory workshops include a broad array of information and topics covered and change slightly from year-to-year, depending on knowledge gained scores and alumni feedback. The current roster of workshops include information on the following: the scientific basis of ADRD, current state of diagnosis and treatments of ADRD, social determinants of health and their relation to ADRD health inequities, the nurse’s role in ADRD risk reduction, how to communicate evidence-based messages with lay audiences, how to identify and combat mis- and dis-information, how to set up public speaking events, and more (see Figure 1 for Brain Health Equity Nurse Fellowship workshop titles and program components). Additionally, starting in cohort #3, all peers were eligible to receive Continuing Nursing Education (CNE) credits for their participation, and in cohort #4, all fellows were eligible to receive CNE credits for their participation in the virtual workshops.

To date, the program has reached 1,093 people: 33 Fellows have completed the fellowship program, and those fellows have reached 503 peers and 557 community members with community conversations focused on ADRD risk reduction. Following the community conversations, 98% of peers feel comfortable sharing information learned with patients (Cohort 1 data), and 85% of community members increased their knowledge of ADRD risk reduction through lifestyle changes (Cohort 2 data). The fellows themselves have commented on the positive impact the fellowship has had on them, personally and professionally. The following two quotes come from fellow exit interviews conducted by an external evaluator:

The number of resources that this fellowship put at my disposal, it’s unbelievable.

Knowing the information we know now, this is going to change how we approach the needs of our patients and how we connect with them, in whatever specialty of nursing.

### Outreach tools for community-based organizations

UsA2 used the data collected during the initial fact-finding activities (landscape assessment and focus groups) to produce “A Practical Guide: Communicating Brain Health Messages with Latino and African American Communities” (Practical Guide) which was co-branded by CDC and later translated into Spanish. The guide identifies best practices for delivering messages to African American and Latino communities and gives tips on creating appropriate, culturally relevant content written at a literacy level that is accessible to all. In turn, the guide informed a social media toolkit that provided social media content for community-based organizations. Drafts of both the Practical Guide and toolkit were reviewed by community-based organizations, a process led by Alzheimer’s LA, to ensure that the final documents best addressed the specific needs of community-based organizations communicating brain health messages to African American and Latino communities.

The Center distributed the guide and social media toolkit to approximately 400 recipients, primarily individuals at community-based organizations, nonprofits working with priority populations, research institutions, and other health care professionals. Between 2020 and April 2024, users have downloaded the guide 386 times and the toolkit 96 times.

To increase the dissemination of messages curated in the Practical Guide, UsA2 reformatted it as a presentation, which has been given at several events (the 2023 Gerontological Society of America annual meeting, the 2024 American Public Health Association annual meeting) and used by partners for their meetings. Partners like the Memory Impairment and Neurodegenerative Dementia (MIND) Center adapted the guide for use during the 2022 Alzheimer’s Mississippi State Planning Summit with key state planning stakeholders and during the 2023 Mississippi Brain Health Inequities Symposium, which included more than 200 attendees, with nurses and social workers in attendance receiving continuing education credits.

UsA2 also developed and deployed culturally tailored educational workshops and trainings. UsA2 adapted a publicly available CDC public health module into an online course called “Understanding Inequities in Alzheimer’s & Other Dementias.” This course was the first course offered during the inaugural Brain Health Academy (a series of free online classes for providers, launched yearly) in June 2022 where 471 people attended (215 of whom received continuing education credits for their participation). Dr. Adriana Perez of the University of Pennsylvania and Ms. Stephanie Monroe led the course. UsA2 also incorporated this course as one of five mandatory workshops that participants completing the Brain Health Equity Nurse Fellowship are required to attend.

### Working with community-based organizations to disseminate messages about risk reduction in priority populations

Alzheimer’s LA used the “Practical Guide: Communicating Brain Health Messages with Latino and African American Communities” to inform two “Ask Me Anything” live Instagram videos, one each for Latino and African American people in the greater Los Angeles region. Messages during the “Ask Me Anything” sessions focused on risk reduction and brain health promotion strategies and reached 316 people.

Additionally, UsA2 and partners (NAHN, NBNA, Alzheimer’s LA, and the MIND Center) created evidence-based brain health social media messages, informed by the Practical Guide and social media toolkit, reaching approximately 300,000 individuals in Grant Year 3 (2022–2023; the latest year the data are available). UsA2 tracked much of this information through reports from partners and the hashtag #BrainHealthEquity which was used in part to track social media engagement. UsA2 utilized other social media initiatives to reach the priority populations, including creating customized content for events like Black History Month, Minority Health Month, and Hispanic Heritage Month and engaging in tweet chats (online conversations that bring several organizations together over Twitter/X to engage organizations and users in a discussion regarding a specific topic) with external groups (such as Salud America), which generated over 4 million impressions.

### Sharing evidence-based information directly to priority populations using digital and social media strategies

**Figure 2.**
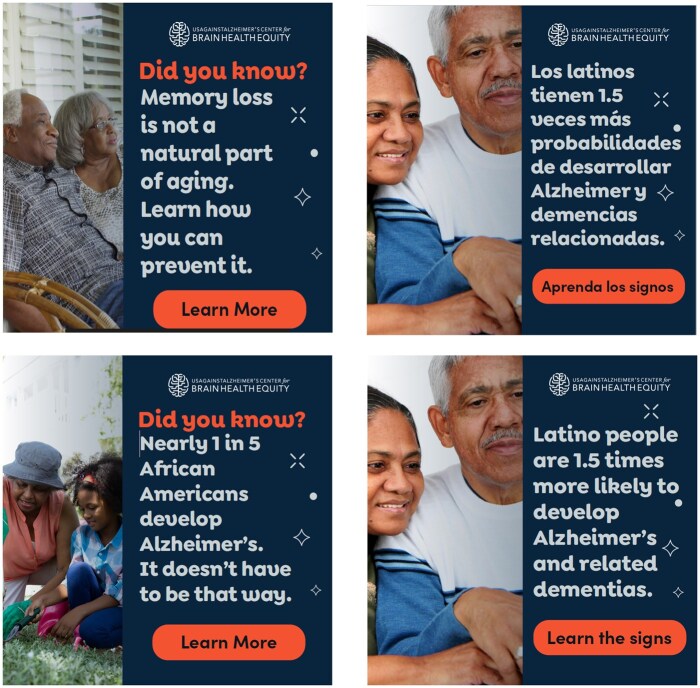
Sample ads used during 2024 ad campaign.

UsA2 developed digital ads, tailored for African American and Latino viewers, in both English and Spanish, for placement on sites and social media platforms. Between 2021 and 2024, each digital ad campaign ran for about 2 months per year during the summer to take advantage of lower ad costs (late summers tend to be the most economical times of year for purchasing online ads). The language and images used in the ads slightly differed from year to year but, overall, the ads focused on simple, relatable brain health messages, focused on themes that resonate with the priority population (as identified during our landscape analysis and outlined in the Practical Guide). Images were intentionally chosen to reflect the reality of these priority populations—such as using a range of skin tones for the Spanish-language, Latino-focused ads; and using different depictions of families, beyond dyads, for both priority populations. See Figure 2 for sample ads used during the 2024 ad campaign.

When viewers clicked on the “Learn More” link within the ads, they were connected to a customized page on UsA2’s consumer-facing site BrainGuide^®^ (UsAgainstAlzheimer’s, n.d), where visitors could find evidence-based prevention and brain health information tailored to specific priority populations, including African Americans, Latinos, and women. The site is available in English and Spanish.

From 2020 to 2023, the campaign’s primary goal was “reach”, as defined by how many individual African American and/or Latino people *see the ad*. The “reach” campaign placed ads on sites that people from the priority populations disproportionately visited, such as theroot.com. In 2024, UsA2 changed the primary goal of the campaign from a “reach” to an “engagement” campaign, as defined by how many Black and/or Latino viewers *click on the ad* to get more information. The engagement campaign strategy did not place ads on specific sites, rather the ads followed the user around to different, unspecified websites with the intention that eventually the user sees the ad enough times to decide to click on it.

Additionally, 2020–2023 reach campaigns had a national reach, while the 2024 engagement campaign was focused in 13 key markets (by zip code and/or county) that have a disproportionate overrepresentation of ADRD in older African Americans and/or Latinos, as identified by the National Alzheimer’s Disease Index (NADEX; a proprietary UsA2 database that analyzes Medicare fee-for-service data about Alzheimer’s disease). While UsA2 reached 48,810 fewer people in 2024 (compared to 2023), there were 290,464 more clicks to the landing page, proving that the engagement strategy was more effective at reaching priority populations, even when using far fewer markets.

**Figure 3.**
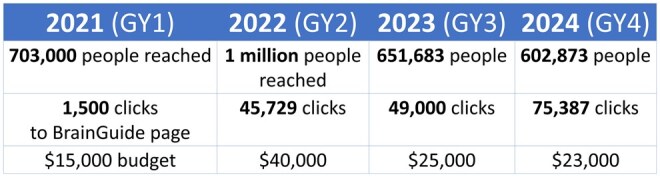
Digital ad campaign summary. The columns list the years that the 2-month campaign took place. Row 1 shows “reach results” (how many individual African Americans and/or Latinos saw the ad). Row 2 shows “engagement results” (how many clicks were made). Row 3 shows the spending budget used for the campaign that year.

In total, from 2020 to 2024, the digital ad campaign has reached approximately 3 million African American and Latino people, and 435,693 African American and Latino users have clicked an ad for more information (see Figure 3 for a summary of the digital ad campaign).

### Educating policymakers about brain health

The final area of activities to increase awareness among priority populations involved educating policymakers about the role of public health in promoting brain health. UsA2 used NADEX to prepare several materials made available to federal policymakers, such as customized congressional district–level reports on the financial impact of ADRD on Medicare beneficiaries, congressional briefings about the role of public health in Alzheimer’s, and a report summarizing the impact of Alzheimer’s on communities of color (the top 50 congressional districts where African American and Latino people are most impacted by Alzheimer’s). UsA2 made these materials available to all members of Congress, but efforts also focused on members of the Congressional Black Caucus, Congressional Hispanic Caucus, and Congressional Alzheimer’s Task Force.

### Next steps

Based on the lessons learned, future research and activities should focus on scaling the Fellowship and addressing remaining gaps through increased digital engagement strategies. More research to formally evaluate long-term behavior change and effectiveness should also be prioritized.

## Conclusion

To address the growing number of African American and Latino individuals in the United States at risk of or living with ADRD, it is crucial for the public health system to adopt strategies for effective communication with these communities. There is a common misperception that these populations are challenging to reach or unresponsive to health care providers.

Successfully engaging with African American and Latino individuals requires the use of proven, validated, and effective strategies, with three key principles in mind: Messages must be (1) culturally relevant, (2) presented at an appropriate health literacy level, and (3) delivered by trusted messengers through channels known to resonate within the community.

By focusing on culturally tailored messaging, empowering trusted healthcare professionals, like nurses, and leveraging digital platforms, the program has made strides in increasing awareness and knowledge about brain health. The multifaceted approach employed by UsAgainstAlzheimer’s, in partnership with organizational partners and the CDC, has proven that reaching African American and Latino communities is possible with intentional outreach.

## Data Availability

This article does not report data and therefore the pre-registration and data availability requirements are not applicable.
